# Using Geographically Weighted Regression (GWR) to Explore Spatial Varying Relationships of Immature Mosquitoes and Human Densities with the Incidence of Dengue

**DOI:** 10.3390/ijerph8072798

**Published:** 2011-07-06

**Authors:** Chia-Hsien Lin, Tzai-Hung Wen

**Affiliations:** Department of Geography, College of Science, National Taiwan University, Taipei 106, Taiwan; E-Mail: chlin1983@gmail.com

**Keywords:** dengue, spatial heterogeneity, geographically weighted regression (GWR), human density, *Aedes* mosquitoes

## Abstract

The only way for dengue to spread in the human population is through the human-mosquito-human cycle. Most research in this field discusses the dengue-mosquito or dengue-human relationships over a particular study area, but few have explored the local spatial variations of dengue-mosquito and dengue-human relationships within a study area. This study examined whether spatial heterogeneity exists in these relationships. We used Ordinary Least Squares (OLS) and Geographically Weighted Regression (GWR) models to analyze spatial relationships and identify the geographical heterogeneities by using the information of entomology and dengue cases in the cities of Kaohsiung and Fengshan in 2002. Our findings indicate that dengue-mosquito and dengue-human relationships were significantly spatially non-stationary. This means that in some areas higher dengue incidences were associated with higher vector/host densities, but in some areas higher incidences were related to lower vector/host densities. We demonstrated that a GWR model can be used to geographically differentiate the relationships of dengue incidence with immature mosquito and human densities. This study provides more insights into spatial targeting of intervention and control programs against dengue outbreaks within the study areas.

## 1. Introduction

Dengue is the most rapidly spreading mosquito-borne viral disease in the World [1]. Its incidence has increased 30-fold in the last 50 years and has extended to new areas, across both rural and urban environments [1]. The South, South-East Asia and the Western Pacific WHO regions are among the most affected areas [1]. Dengue or dengue-like transmission has been observed in southern Taiwan since the late 19th century [2], initially as intermittent epidemics at intervals of up to 40 years [3]. However, for the past 10 years, dengue epidemics have become an annual phenomenon with the cities of Kaohsiung and Fengshan as the main foci of activity.

The dengue viruses (DENVs) are transmitted to humans by *Aedes (Ae.)* mosquitoes (vector), in particular *Ae. aegypti* and *Ae. albopictus* [1,4]. There is currently no effective treatment or available vaccine against dengue [5], hence current prevention and control policies mainly aim to reduce human-mosquito contact or to decrease the vector population to levels where viral transmission is unsustainable. To ensure efficient prevention policies, it is important to understand the relative impact of vector and host density on the dispersal of DENVs within an area.

The relationships of the dengue incidence–mosquito abundance and dengue incidence-human density are still not well understood. Since the density of adult mosquitoes is difficult to estimate, immature vector data were widely used for evaluating the incidence–mosquito relationship [6–14]. Some entomology studies have found no correlation between dengue incidence and immature vectors, neither temporally nor spatially [6–10]. However, a study in Trinidad recently showed that high dengue incidences were significantly related to high mosquito larval densities during certain years [11]. Other spatial studies in Cuba, Trinidad and Thailand have successfully demonstrated that the Breteau index (BI) and house index can be an indicator for incidence [12–14]. The association of dengue incidence and human density are also ambiguous. Studies in Brazil have shown no correlation between dengue incidence and human density [15], but researches in both Taiwan and Puerto Rico have proven that the spatial distribution of dengue incidence may be positively related to the population density [16,17]. Population density and urbanization are also considered as risk factors for DENV spread in Argentina and Hawaii [18,19]. Moreover, in hyperendemic areas of Thailand, DENVs transmission is more prevalent in children in localized neighborhoods [20].

Until now, most studies of dengue-mosquito or dengue-human relationships have presented a global perspective by which any relationship was assumed to be spatially constant across the whole study area, thereby ignoring local variations. However, this assumption may be inappropriate since the dengue-mosquito or dengue-human relationships could be positively correlated in some study areas, but negatively or not correlated at all in other areas. For example, a small number of female mosquitoes in a very dense area is sufficient to cause an outbreak. This study was conducted to evaluate the hypothesis that spatial heterogeneity existed for dengue-mosquito and dengue-human relationships. We demonstrated that the variation of dengue incidences among study areas was reflected by the densities of both immature vectors and hosts. By capturing the local relationships across the space, the authorities can design more effective, locally-specific strategies. This understanding is especially important where the control and prevention resources are limited.

## 2. Materials and Methods

### 2.1. Study Area

**Figure 1:**
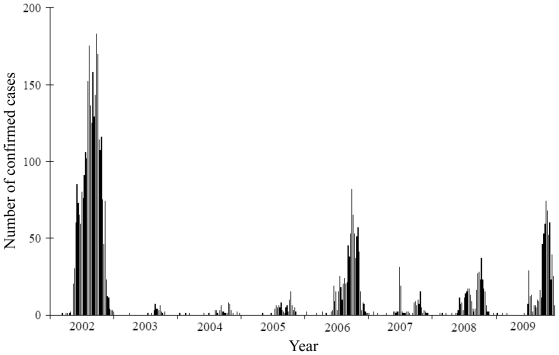
The epidemic curve of confirmed dengue cases as cumulated by weeks of onset in Kaohsiung and Fengshan cities, 2002–2009.

Kaohsiung city has been the epicenter where most of the dengue outbreaks have been recorded in Taiwan [3]. It is a major port and industrial metropolis with a population of some 1.5 million. Kaohsiung International Airport is an important access point for visitors as well as foreign workers, many of whom are employed in the city’s commercial harbor. The large industrial and export processing zones of Kaohsiung city also attract around 15,000 foreign workers per year, mainly citizens from neighboring countries such as Philippines, Indonesia, Vietnam and Thailand. Kaohsiung city, which covers a total area of 150 km^2^, is the most densely populated urban centre in Taiwan. The neighboring Fengshan city, located directly to its east, has a population of 330,000 within an area of 27 km^2^. Piped water is available for 99% of the city households and household waste is removed daily throughout both cities by the government. The 2002 dengue epidemic in which Kaohsiung and Fengshan cities were the major foci was the largest outbreak in recent years in Taiwan (Figure 1), with more than 15,000 reported cases, with a total of 3,786 confirmed dengue cases [3].

**Figure 2:**
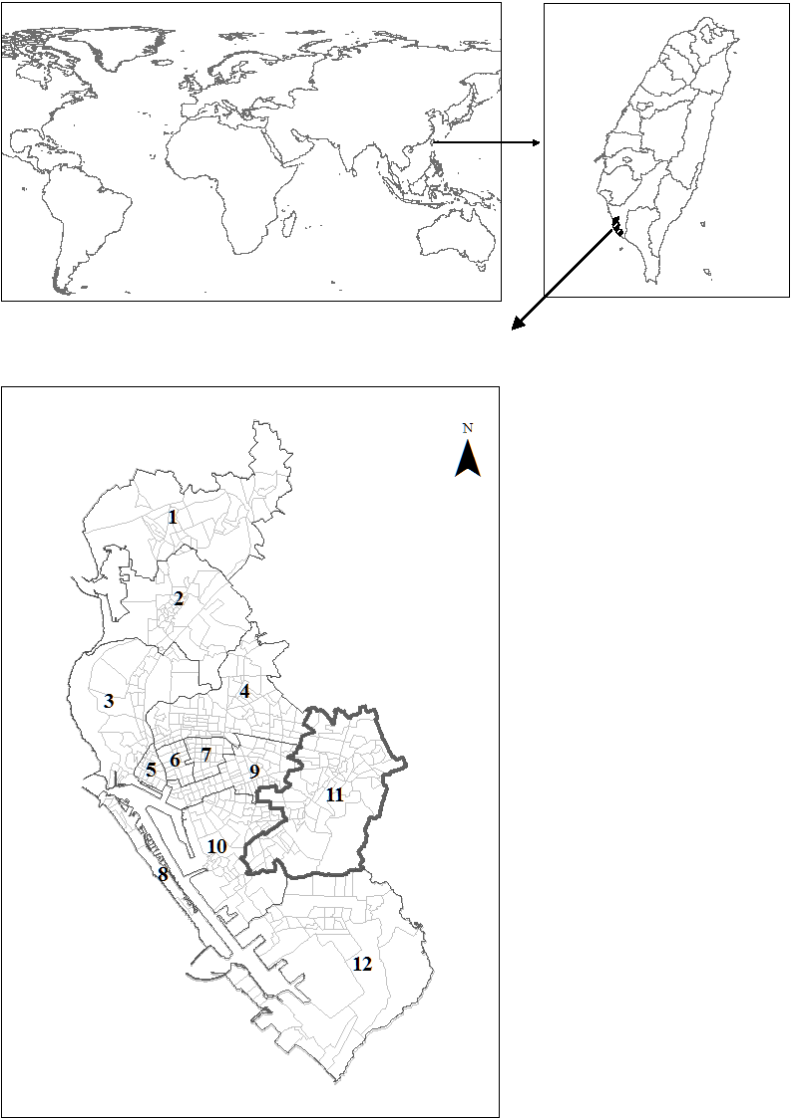
The distribution of 542 Lis and 12 districts in Kaohsiung (district 1–10 and 12) and Fengshan cities (district 11) in Taiwan. Each small polygon represents each Li.

The “Li”, the lowest administrative unit in Taiwan, was used as the spatial mapping unit in this study. There were a total of 542 Lis and 12 districts in the two cities during the study period of 2002. On average each Li had a population of 3,366 and 1138.41 households in an area of 0.36 km^2^. The study area is shown in Figure 2.

### 2.2. Dengue Data

All information on dengue cases was provided by the Centers for Disease Control-Taiwan (Taiwan CDC). Laboratory confirmation was obtained for all suspected cases identified through passive, active and passive-based active surveillance activities. Passive surveillance involved the mandatory referral of suspected dengue cases within 24 h of presentation at any of 231 health clinics or hospitals (both private and public), school-based reports of absence due to fever as well as individual self-reporting [3]. Self-reporting refers to any requests for a free dengue test by patients presenting at any public health center. Active surveillances were maintained through fever checkpoints at the Kaohsiung airport. Passive-based active method was systematic screening of contacts (family members, colleagues and neighbors) of confirmed cases [3]. Laboratory diagnosis of suspected cases was conducted by the fifth branch office of Taiwan CDC in Kaohsiung City. Confirmation of dengue infection using patient serum was obtained by: (1) detection of DENV-specific IgM or IgG by capturing enzyme linked immune-sorbent assay (ELISA) in single sample or fourfold IgG titer increase in paired acute and convalescent samples, or (2) detection of DENV RNA by reverse transcriptase polymerase chain reaction (RT-PCR) [3]. All test expenses were covered by the National Health Insurance.

### 2.3. Immature Mosquito and Human Density

Larval habitats of *Ae. aegypti* and *Ae. albopictus* were observed on a routine basis by trained personnel from the Kaohsiung city Health Bureau and county governments during the study period. All personnel had received training in mosquito species distinction, mosquito habitat recognition techniques and sampling methods. According to the control and prevention protocol of Taiwan CDC, 50 households in each Li were randomly selected for inspection, which covered indoor and outdoor areas of the selected premise. On average, each Li was surveyed once per month. Containers with immature *Ae.* mosquitoes (larvae/pupae) were considered as positive containers [3]. For habitats with low water volume (<30 L) the larvae/pupae would be strained off and transferred into white bowls for visualization and counting. For habitats containing high water volume, as many larvae/pupae would be collected as possible [3]. The stage of larval maturation (1–4 instars) was not documented. The mosquito species was determined following adult emergence from collected specimen reared at the laboratory facilities of the health bureau of Kaohsiung city and county governments.

Breteau index was used to estimate the density of immature *Ae.* mosquitoes in the study. BI is defined as number of positive containers per 100 houses [1], and was estimated on a monthly basis in each Li. The average number of people per unit area (people/km^2^) was taken from 2002 census data as an indicator of human population density (POPden) estimated for each Li.

### 2.4. Statistical Analysis

In this study, the dengue annual cumulative incidence (IR), given as cases per 100,000 populations, was used as the measure of disease severity, and as the dependent variable; independent variables were POPden and the monthly maximum BI detected in each Li in 2002 (BI_max_). A summary of the variables in both Ordinary Least Squares (OLS) and Geographically Weighted Regression (GWR) models are shown in Table 1. We first applied OLS regression, in an attempt to explain the global relations between dependent and independent variables. The model was set as: IR = β_0_ + β_1_ BI_max_+ β_2_ POPden + ɛ. β_0_ and β_1_ were the regression coefficients whereas *ɛ* was the model random error.

The diagnoses of an OLS model were approached by assessing multicollinearity and the residuals. The multicollinearity was assessed through variance inflation factor (VIF) values, and if VIFs were greater than 10, this indicated multicollinearity existed [21]. The spatial independency of residuals was evaluated by the spatial autocorrelation coefficient, Moran’s *I*, which was expressed as:

$$I=\frac{n\sum_{i=1}^{n}\sum_{j=1}^{n}w_{ij}(y_{i}-\bar{y})(y_{j}-\bar{y})}{\left(\sum_{i\neq j}\sum w_{ij}\right)\left(\sum_{i=1}^{n}{\left(y_{i}-\bar{y}\right)}^{2}\right)}$$

where *n* was the total number of Li in the study [19]. *i* and *j* represented different Lis. *y_i_* was the residual of *i*, and *ȳ* was the mean of residuals. *w_ij_* was a measure of spatial proximity pairs of *i* and *j* [22]. We used the inverse of the distance between *i* and *j* for specifying the relationship between them.

The values of Moran's *I* would be approximately between +1 (positive autocorrelation) and −1 (negative autocorrelation), and the expected value in the absence of autocorrelation was (−1)/(n−1). Positive spatial autocorrelation meant similar values tended to occur in adjacent areas, while negative autocorrelation implied nearby locations tended to have dissimilar values. If no spatial autocorrelation was found, then the spatial arrangement would be completely random [23].

A GWR local model was applied to analyze how the IR-BI_max_ and POPden relationships changed from one Li to another. It was a localized multivariate regression that allowed the parameters of a regression estimation to change locally. Unlike conventional regression, which produced a single regression equation to summarize global relationships among the independent and dependent variables, GWR detected spatial variation of relationships in a model and produced maps for exploring and interpreting spatial non-stationarity [24]. GWR was calibrated by multiplying the geographically weighted matrix *w(g)* consisting of geo-referenced data [24,25]. The *w(g)* was defined by the spatial neighboring relations between points, which can be presented as:

$$w(g)=\left[\begin{array}{ccccc} w_{g1} & 0 & 0 & \ldots & 0\\ 0 & w_{g2} & 0 & \ldots & 0\\ 0 & 0 & w_{g3} & \ldots & 0\\ . & . & . & \ldots & .\\ 0 & 0 & 0 & \ldots & w_{gn} \end{array}\right]$$

Within the matrix, *w_gn_* referred to the impact between position *g* and position *n* in which the values range between 0 and 1. This study presumed the degree of impact had an inverse ratio to the square distance of different Lis. In other words, the larger the *w_gn_* was, the closer geographically data points were, and the stronger impact they had on each other.

The spatial variability of an estimated local regression coefficient was examined to determine whether the underlying process exhibited spatial heterogeneity [24,25]. The regression model can be rewritten as IR*_i_*(g) = β_0_*_i_*(g) + β_1_*_i_* BI_max_*_i_*(g) + β_2_*_i_* POPden*_i_*(g) + ɛ*_i_*, where (g) indicated the parameters that were estimated at each Li in which the coordinates were given by vector g; *i* represented each Li. By applying GWR modeling, the spatial influences among neighborhoods could be assessed, which was not able to be achieved through traditional OLS methods [25].

We also examined the local collinearity as well as the independency and normality of residuals of GWR model to evaluate the fit of the model. The local collinearity was assessed by scatter plots of the local coefficient estimates for BI_max_ and POPden and condition number. The condition number is the square root of the largest eigenvalue divided by the smallest eigenvalue. If the condition numbers are greater than 30, multicollinearity would be a very serious concern. The adjusted coefficient of determination (Adjusted R^2^), and ANOVA were used for comparing OLS and GWR models. Akaike Information Criterion (AIC) generated for OLS and corrected Akaike Information Criterion (AICc) calculated for GWR were also used for model comparison [24]. The concept here is to determine which model could interpret data better.

Our analysis in this article was based on Li-level data. All analyses were implemented using ESRI^®^ArcGIS^TM^9.3 and GWR 3.0 with 0.05 significance level. In the GWR model, the adaptive kernel with AICc estimated bandwidth was chosen. The adaptive kernel was chosen because the distribution of Li was inhomogeneous in the study area (Figure 2). The data set from the 2002 dengue outbreak in Kaohsiung and Fengshan cities was provided by Taiwan CDC. We aggregated the confirmed dengue cases with home address to each Li for regression analyses.

## 3. Results

### 3.1. OLS Regression

**Figure 3:**
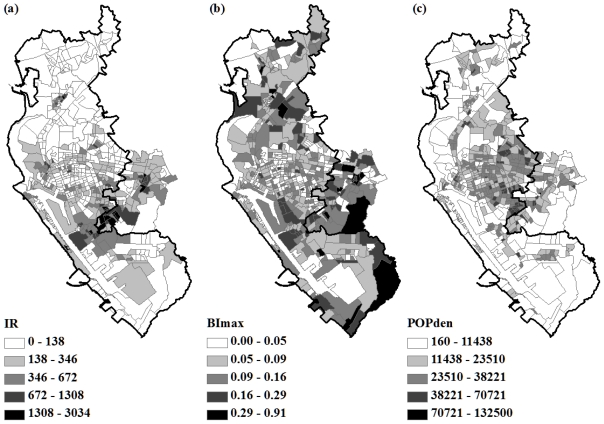
Spatial distributions of (**a**) dengue incidence (IR); (**b**) maximum Breteau index (BI_max_); and (**c**) population density (POPden) in each Li in Kaohsiung and Fengshan cities, 2002. IR was based on 2002 census with the unit of case per 100,000 population. The unit of POPden was populations per km^2^. Li was the basic administrative unit in Taiwan, and there were 542 Lis in the study area.

The spatial distributions of the IR, BI_max_ and POPden were mapped in Figure 3. The map of cumulative IR showed high values clustered in some border areas. The northern areas generally had lower IR values than middle and southern areas in the cities. The pattern of maximum Breteau index was less obvious. High population density was found in both city centers.

The results of applying OLS regression showed that holding the variable of population density fixed, ceteris paribus, one BI_max_ increase is significantly associated with 947.93 increase of average IR (Table 2). The VIF values indicated OLS estimations were not biased from multicollinearity. However, this global regression model explained only 4 percent of the total variance of IR with the AIC 7,902.12. We further examined the residuals of the OLS model, and found the residuals had positive spatial autocorrelation (Moran’s *I* = 0.28, *p* < 0.01). Since the existence of dependent residuals violates the assumptions of OLS estimation, we employed a GWR model to fit the data. We used GWR to present the spatial diversities of the IR-BI_max_ and POPden relationships.

### 3.2. GWR Model and Spatial Variations

The summary results of GWR are listed in Table 3 and showed the GWR was more suitable than the OLS model since GWR could explain 59 percent of the total model variation with the decreased AICc. Moreover, the ANOVA comparison results also showed the GWR local model was significantly more appropriate than the OLS global model (F = 5.36, *p* < 0.001).

**Figure 4:**
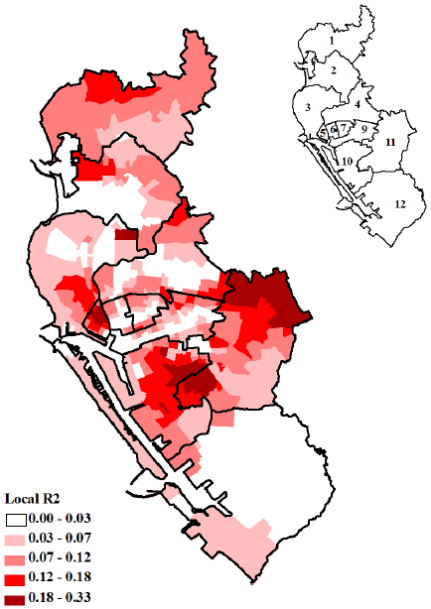
Spatial mapping of the locally weighed coefficient of determination (R^2^) between the observed and fitted values by geographically weighted regression (GWR) modeling. The data presented here were the 2002 dengue incidence, the maximum Breteau index and population density in each Li in Kaohsiung and Fengshan cities.

**Figure 5:**
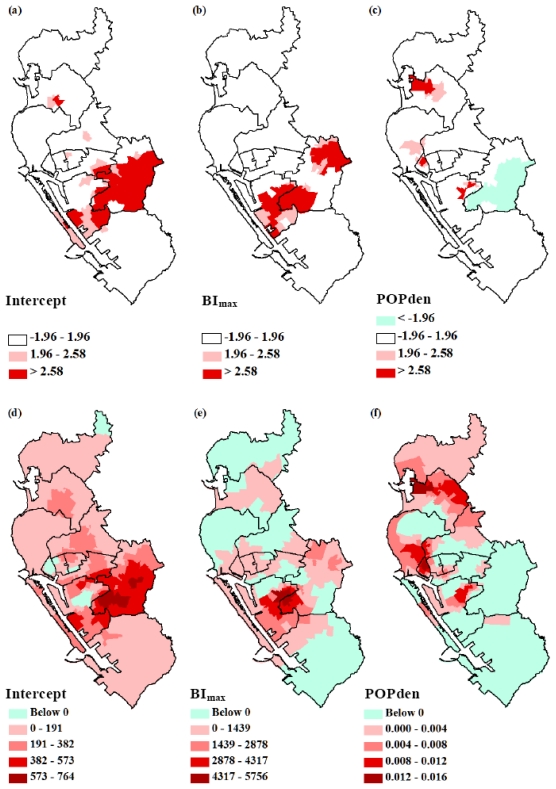
Spatial mapping of pseudo *t* values of regression fitting (a–c) and the coefficients (d-f) of intercept, maximum Breteau index (BI_max_) and population density (POPden) for each Li by geographically weighted regression (GWR) modeling. The dependent variable was dengue incidence (per 100,000 populations) taken from 2002 dengue epidemic data in Kaohsiung and Fengshan cities. Each polygon represents each district.

Figure 4 showed the maps of the locally weighed R^2^ between the observed and fitted values, which indicated how well the GWR model replicated the local IR around BI_max_ and POPden. It was obvious that the value of R^2^ was not homogeneously distributed in all Lis, and the overall GWR regression fitted best in districts 1, 5, 10 and 11. This model did not fit well in district 12, and this could imply additional covariates were needed to explain the IR in district 12. Figure 4 helped us realize whether additional explanatory factors were required and where could those factors be applied. We also mapped the pseudo *t* values for intercept and each dependent variable to represent the fitting level for each specific variable under GWR [Figure 5(a)–(c)]. The significant *t* values, blue and red areas, indicated that the parameter estimations in these areas were reliable.

**Figure 6:**
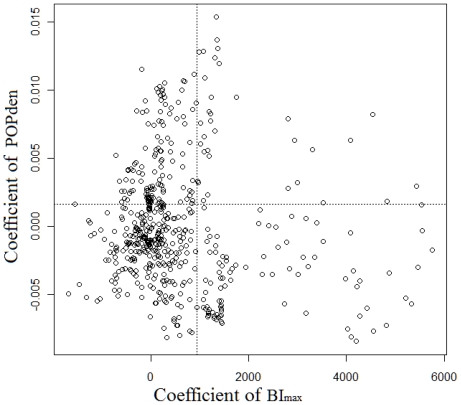
Scatter plot of the GWR coefficients of population density (POPden) and maximum Breteau index (BI_max_) with R^2^ = 0.01. The dashed lines were the levels of the OLS estimations.

**Figure 7:**
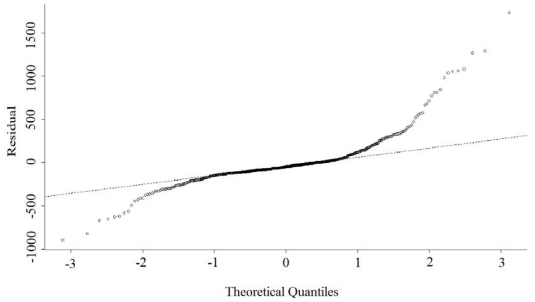
Normal quantile-quantile plot of the residuals from GWR estimations.

The condition number shown in Table 3 and the scatter plot of the GWR coefficients suggested multicollinearity was not serious (Figure 6). However, the local residuals deviated, since residuals showed moderate positive spatial autocorrelation (Moran’s *I* = 0.02, *p* = 0.02), and some parts failed to follow a normal distribution (Figure 7).

**Figure 8:**
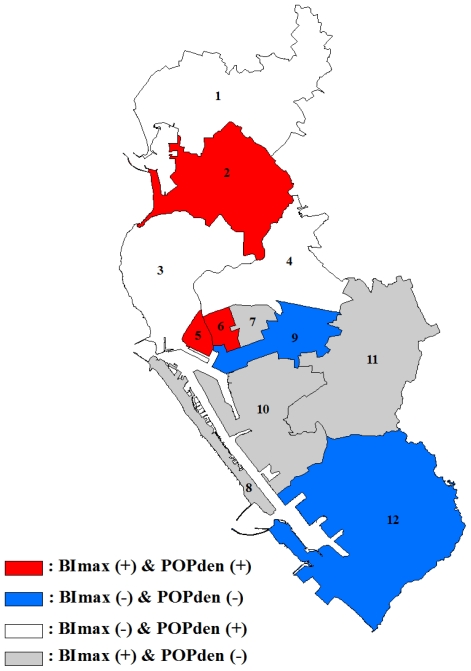
Summary of BI_max_ and POPden impact on incidence using GWR in each district. BI_max_ (+) means BI_max_ had positive impact on incidence while BI_max_ (−) means BI_max_ had negative impact on incidence; POPden (+) means POPden had positive impact on incidence whereas POPden (−) means POPden had negative impact on incidence.

The spatial variations in parameter estimations for intercept, maximum Breteau index and population density are shown in Figure 5(d)–(f). The map of intercept term represented the distributions of IR when BI_max_ and POPden equaled zero. It was observed that higher intercept values were located around the borders of two cities [districts 9, 10 and 11, Figure 5(d)]. This spatial heterogeneity implied that besides immature mosquito and population density, there were still other variables that would influence IR pattern. The relationship between IR and BI_max_ shown in Figure 5(e) suggested that, *ceteris paribus*, in districts 2, 5, 6, 7, 8, 10 and 11, increased IR would relate to increased BI_max_. However, in the remaining districts, higher IR associated with lower BI_max_ and *vice versa*. The distribution of population density parameter showed a more clearly spatial non-stationary pattern [Figure 5(f)]. The positive relationships that were mostly clustered in the northern areas, indicating higher population density tended to associate with higher IR. On the contrary, the impact of population density on IR was negative in the southern parts representing higher IR related to lower population density. A brief summary of these relationships is shown in Figure 8.

## 4. Discussion

This study provides further indications that the relationships of dengue incidence-maximum BI and dengue incidence-population density were spatially non-stationary in Kaohsiung and Fengshan cities. In regression maps, it is clear that the intensity and directions of the influence of maximum BI and population density on dengue incidence were different in the study area. This result gives the policy makers more ideas how to better adopt specific control and prevention strategies to specific areas [26].

The spatial heterogeneity of intercept results in Figure 5(a) could imply that the DENVs seroprevalence was non-stationary. Our study found that the density of immature vectors was a significant predictor of dengue incidence in some areas with either positive or negative correlations. Reducing immature mosquito densities is currently the major control and prevention approach for dengue [1]. However, the results from this study suggest that this strategy may not be spatially and universally suitable for the control of dengue, especially for those areas with negative incidence-maximum BI correlations. Possible reasons could be that other than immature mosquito density, local characteristics could affect dengue transmission as well. For instance, some rural areas with high vector density may lack common exposure sites to humans, thus making outbreaks less likely to occur. On the contrary, places where crowds gather easily like markets, parks, train stations and schools may propel huge dengue outbreaks even though the mosquito density is low [17]. Human activities that promote host-vector contact increases the risks for people to be infected within a short distance [1,27,28]. This study showed that the distribution of dengue incidence-BI relationships was very similar to the distribution of districts which implied the presence of additional risk factors, such as the age distribution in human population, human activity [15,29], housing structures/patterns [30,31], environmental factors [29], and serosurveillance [32]. These other factors should also be considered, since the diversities of these factors were large among the districts.

In the northern part of the study area, higher human densities were shown to contribute to higher dengue incidence rates. This positive relationship was expected as higher human density may lead to higher vector-host contact rates. A previous finding in Taiwan showed that the relative risk of accumulated dengue incidence for areas with more than 10,000 people/km^2^ was 10-fold compared to areas with less than 1,000 people/km^2^ [16]. Other studies in Florida and Puerto Rico showed that the human population had almost the same spatial pattern as the number of dengue cases during the study period [17]. However, the GWR results also demonstrated that in some areas higher incidence related to lower human densities and vice versa [Figure 5(f)]. One explanation could be that in scattered populated areas, mosquitoes tend to aggregate since fewer blood sources were available [33]. Human travelling behaviors should also be taken into account for the link between higher incidence and lower population density. Travelers not only could initiate new indigenous epidemics, their travelling waves could also contribute to dengue occurrence in low population and rural areas [34,35]. According to our findings and those from other studies mentioned above, the relationship between human population/density and dengue occurrence remains controversial. Further studies should take more spatial information into consideration such as dwelling density [31], type of household [36,37], socioeconomic status [15,31], age and gender distributions [31], pesticide spraying areas and frequencies, water storage habits and landscape [37,38]. Successful dengue transmission requires that the virus, vector, and host exist in the same areas and interact properly. Understanding the relationship among them is necessary and urgent for more effective disease control.

The relationship between vector abundance (both immature and mature stages) and dengue occurrence has been discussed in many studies [6,8,39]. This is a practical issue especially important for policy makers to decide the control and prevention measures. This study provides insight into the spatial heterogeneity of IR-immature mosquito density relationships at Li level; however, there were some limitations for applying entomology data. First, the traditional indicators (*Stegomyia* indices) such as house index, container index and Breteau index are based on the immature stages of mosquitoes, but larvae/pupae quantities have no direct link with adult abundance and thus an estimate of dengue transmission risk may not be reliable [8,40]. Moreover, these indices provide little information about the container productivity of vector. Assuming all positive habitats have equal vector contribution could lead the researchers to make false estimations of adult amount [41]. The information like number of vector per person or per unit area, which also relates to dengue transmission is disregarded in these indices as well [42]. Moreover, if we directly apply adult index for dengue risk assessment to avoid the limitations of immature stage data, the major problem would be the ratio of captured vectors to existing mosquitoes is still unknown. In this study we chose the monthly maximum BI in each Li as the measurement since we assumed the maximum BI was the best entomology indicator for the dengue cases. In addition to the vector indices problems, the susceptibility of the population to a specific dengue virus serotype is also a great contributor to the scale of epidemics. Once infected a person would acquire lifelong protective immunity to the infective serotype [43]; in other words, the incidence estimation is hindered by a lack of information concerning the overall population immunity to certain serotypes. This makes the estimation of case-vector relationship more complicated. Finally, silent DENVs transmission was not considered in this study.

To improve the understanding of incidence-vector and incidence-host relationships, the followings could be further examined. First of all, the researchers could adopt GWR space-time analyses, such as stratifying the year of 2002 into different periods, or analyzing more than one epidemic year. This approach could provide more detailed patterns of spatial autocorrelation changes of incidence-vector and incidence-host associations. Secondly, the researchers could use other BI calculations such as minimum BI or average BI to see whether different incidence-BI relationships would be generated. Threshold effect of BI could also be considered. Thirdly, categorizing human by different age groups in the GWR model could assist policy makers to determine which actions are suitable for different populations. Finally, researchers could also separate *Ae. aegypti* and *Ae. albopictus* for relationship analyses to study the incidences associated with different vector ecologies.

The geographical heterogeneity was detected by the GWR method in the relationships of dengue incidence with immature mosquito and human density (Figure 5). We used GWR since the conventional regression, OLS, cannot discriminate the spatial variation in relationships if geographical nonstationarity exists. The results of Adjusted R^2^, AIC/AICc and ANOVA all indicated GWR was a better model to explain this dataset. GWR approaches have been applied in a lot of areas, such as public health and demography, as an exploring method for identifying the spatial variations [44–46]. However, the GWR applications are limited for some reasons. First, the results conducted from GWR models were very sensitive to the chosen kernel type and bandwidth methods [47,48]. Next, the non-linear term cannot be added in the GWR models and the model inferences cannot be done in GWR [24]. Future research could use more advanced methods like Bayesian additive regression models, which are based on Markov chain Monte Carlo (MCMC) algorithms for parameter estimations and inferences to overcome the problems mentioned above [49].

## 5. Conclusions

This paper underlines the spatial variations of incidence-immature mosquito density and incidence-human density relationships in a local scale. Exploring the heterogeneity of spatial relationships could provide more insights into spatial targeting of intervention against dengue epidemics.

## Figures and Tables

**Table 1:** Summary of dependent and independent variables used in OLS and GWR.

Variable		Numerator	Denominator
Dependent:	IR	100,000 × number of cases	Populations
Independent:	BI_max_	100 × number of positive containers	Number of premises inspected
	POPden	Populations	The area of Li (km^2^)

IR: cumulative incidence of dengue; BI_max_: Maximum Breteau index; POPden: Population density.

**Table 2:** Ordinary Least Squares (OLS) results.

Parameter	Estimated Value	Standard Error	*p*-value	VIF
Intercept	115.52	34.73	0.003	
BI_max_	947.93	202.48	0.013	1.02
POPden	0.00	0.00	0.111	1.02
Adjusted R^2^	0.04			
AIC	7,902.12			

**Table 3:** Geographical weighted regression (GWR) results.

Parameter	Minimum	25% quartile	50 % quartile	75 % quartile	Maximum
Intercept	−272.60	78.46	166.09	320.92	1,088.31
BImax	−2980.43	−262.53	100.40	838.91	5,797.87
POPden	−0.02	−0.00	0.00	0.00	0.02
Condition number	2.96	4.67	5.83	7.32 10.39	
Adjusted R^2^	0.59				
AICc	7,715.17				
